# Distribution of bone fragments in angled shots: an experimental study conducted on composite models containing artificial bone plates

**DOI:** 10.1007/s00414-024-03307-y

**Published:** 2024-08-21

**Authors:** Dorothee Geisenberger, Markus Große Perdekamp, Matthieu Glardon, Jan Kromeier, Stefan Pollak, Annette Thierauf-Emberger

**Affiliations:** 1 Institute of Forensic Medicine, Faculty of Medicine, University of Freiburg, Albertstraße 9, Freiburg, 79104 Germany; 2 Forensic Medicine and Imaging, Institute of Legal Medicine, University of Berne, Bühlstrasse 20, Berne, 3012 Switzerland; 3 Department of Radiology, St. Josef’s Hospital, Sautierstraße 1, Freiburg, 79104 Germany

**Keywords:** Gunshot injury, Angled shot, Splinter, Flat bone, Composite model, Artificial bone

## Abstract

In conventional gunshot injuries to targets containing bone the resulting osseous fragments do not precede but follow the bullet on its further way through adjacent soft tissues. The term “secondary projectiles” for the particles does not appear to be appropriate since they are not believed to have enough energy necessary for creating their own wound channels away from the temporary cavity. Former studies have shown that in angled shots to glass panes the bulk of splinters does not follow the bullet’s trajectory: The majority of the glass fragments, especially the larger ones, move at right angles to the pane shot through. The aim of the presented study was to examine whether osseous fragments behave like glass splinters in angled shots to flat synthetic bone. In this context, it should also be assessed, whether the bone fragments might act as secondary projectiles in rare cases. To answer these questions, test shots were fired to composite models consisting of flat synthetic bone and ballistic gelatin. Pistol cartridges 9 mm Luger were used to fire the shots which were video-documented with a high-speed camera. Afterwards, the composite models underwent CT examination and macroscopic inspection. Video-documentation revealed that the larger bone particles from the perforation site move at a roughly right angle from the osseous sheet into the gelatin, causing an eccentric bulge of the temporary cavity. The smaller bone fragments were also lodged along the bullet’s path, predominantly in the cracks radiating from the permanent wound channel.

## Introduction

Bones have a significantly higher density and lower elasticity than soft tissue [1–3]. The different physical properties have substantial impact on the wound morphology of gunshot injuries. Compared with the penetration of soft tissue, the interaction between bullet and bone is more complex [4]. On the part of the projectile, contact with bony structures can cause deformation and early tumbling [1]. The velocity, construction and weight of the bullet as well as the angle of impact are influencing factors [5].

Regarding osseous tissues, distinction must be made between long and flat bones. The latter ones are particularly interesting from the medico-legal point of view, as the bullet holes may provide information on the direction of shot [6, 7] and the projectile’s calibre [8–10]. Further aspects are the formation of bone particles and their final positions in relation to the bullet’s trajectory. Generally, it is to be expected that bone fragments move in the direction of shot, thus following the bullet as it passes through the soft tissues [1, 11]. According to observations made when angled shots hit glass planes, the majority of splinters, especially the larger ones, move at almost right angles to the pane shot through [12, 13].

The aim of the present study was a systematic investigation of angled shots to flat bone under standardized conditions. For this purpose, the bone was simulated by synthetic material and the surrounding soft material by ballistic gelatin. The distribution and final positions of the bone fragments were documented by means of videography using a high-speed camera and by subsequent CT imaging.

## Materials and methods

Composite models made from ballistic gelatin containing flat synthetic bone plates (3-layer bone simulant made from polyurethane, Synbone^®^, Zizers, Switzerland) having a thickness of 6 mm served as targets for test shots fired with pistol cartridges cal. 9 mm Luger. The bone plates were embedded in gelatin blocks at a 45-degree angle relative to the trajectory to simulate an angled shot. Each gelatin block measured 20 × 25 × 30 cm; the gelatin was prepared from a 10% solution according to current recommendations for experiments in wound ballistics [14, 15]. In total, five composite models were fired at. Additionally, one angled shot was fired to a plate of synthetic bone not embedded in gelatin in order to record the fragment’s behaviour in the less dense medium of air.

The cartridges (MAGTECH 9 mm Luger, 124 GR, CBC Global Ammunition, Minneapolis, MN, USA) were fitted with full-metal jacket round nose bullets. The shooting distance was 5 m and the direction of the shots was orthogonal to the front plane of the gelatin blocks containing the inclined bone plates. According to the manufacturer’s specifications, the muzzle velocity of the bullets is in the order of 338 m/s.

All test shots were video-documented using a high-speed motion camera (Photron FastCam SA-X2, San Diego, CA, USA; frame rate up to 13.500 fps) with a view perpendicular to the direction of fire.

The specimens were examined on a 64-MDCT scanner (SOMATOM Definition AS; Siemens Medical Solutions, Forchheim, Germany). Examination parameters: 120 kV, 400 mAs and 140 kV, 600 mAs; 0.6 mm primary collimation; pitch 0.75 mm; system software Syngo CT VB20A. To maximize image quality all dose-saving parameters were shut off. Data was reconstructed using a high-contrast kernel (Br32).

Standard secondary multiplanar reformation was done in 0.6 and 1 mm slide width in two different aspects with a relatively narrow (center/width: 40/300) and a wider window (c/w: -50/1000), respectively.

The VRT (Volume Rendering Technique) reformations were reconstructed using a specialized 3D suite (syngo.via, Siemens Medical Solutions, Forchheim, Germany; software version VB40B).

Subsequent to the CT examination, the gelatin blocks were cut into 1 cm-slices along the whole bullet track and transverse to its longitudinal axis so that a layer-wise localization of the bone fragments was possible.

## Results

In all test shots, the composite models were perforated in full length. The bullets remained intact and suffered only minor deformation. The gunshot holes in the synthetic bone plates were roundish-elliptic with the typical broadening in the direction of shot.

**Fig. 1:**
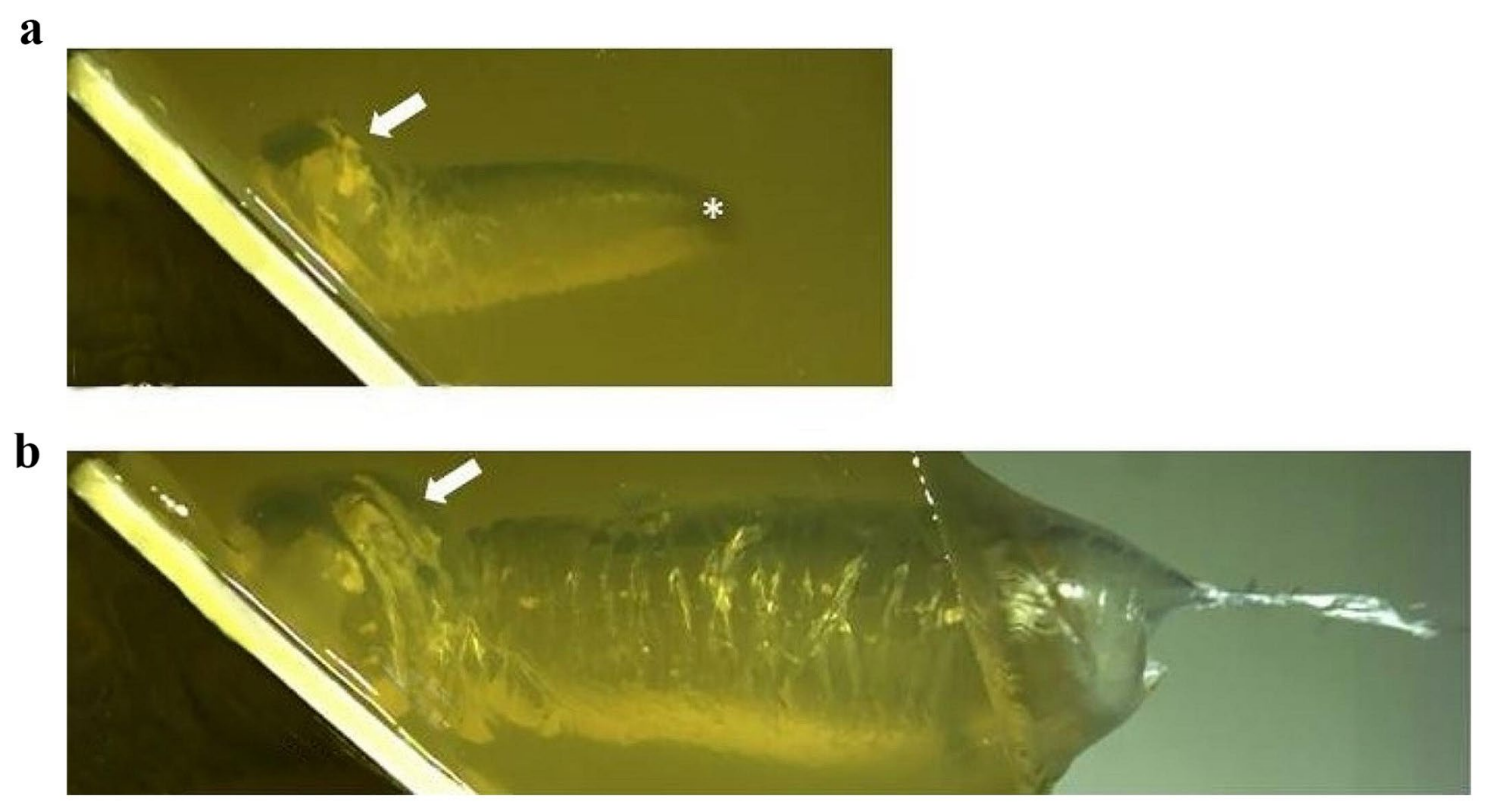
High-speed images of an experimental shot showing the development of the temporary cavity. The *arrows* point to an excentric bulge of the temporary cavity caused by the osseous fragments moving at right angles from the perforated synthetic bone sheet into the gelatin. **a** The *asterisk* indicates the position of the bullet. **b** The projectile has already left the composite model

The temporary cavity developing along the bullet path could be observed by videography due to the transparency of gelatin (Fig. 1a, b). Immediately after the projectile’s passage through the composite model, the temporary cavity had a roughly tubular shape with a diameter exceeding the bullet’s caliber several times.

The bone particles which originated at the bullet hole were transferred to the expanding temporary cavity, but did not create separate wound channels. A bulk of major fragments was deposited in close vicinity to the beveled exit side of the perforated plate. The particles were not distributed along the bullet’s trajectory, but excentrically at a right angle to the plate (Fig. 1a, b). Smaller splinters on the other hand, also travelled for greater distances following the trajectory of the bullet. They often got stuck at the ends of the cracks radiating from the bullet path.

**Fig. 2:**
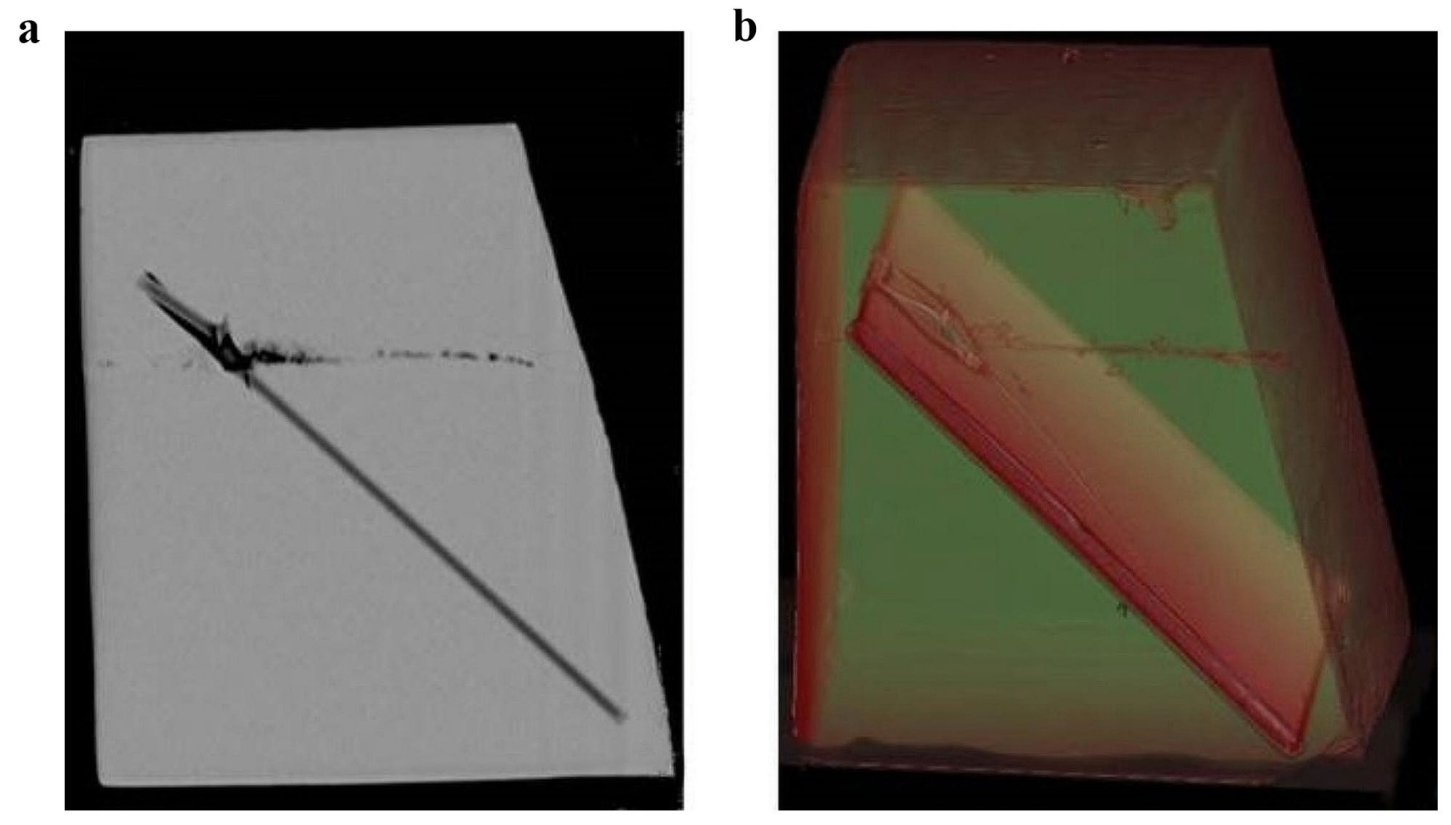
**a** Sagittal reformation in 1 mm slide width using a high contrast kernel and soft tissue window. **b** 3D VRT reformation (oblique side view)

**Fig. 3:**
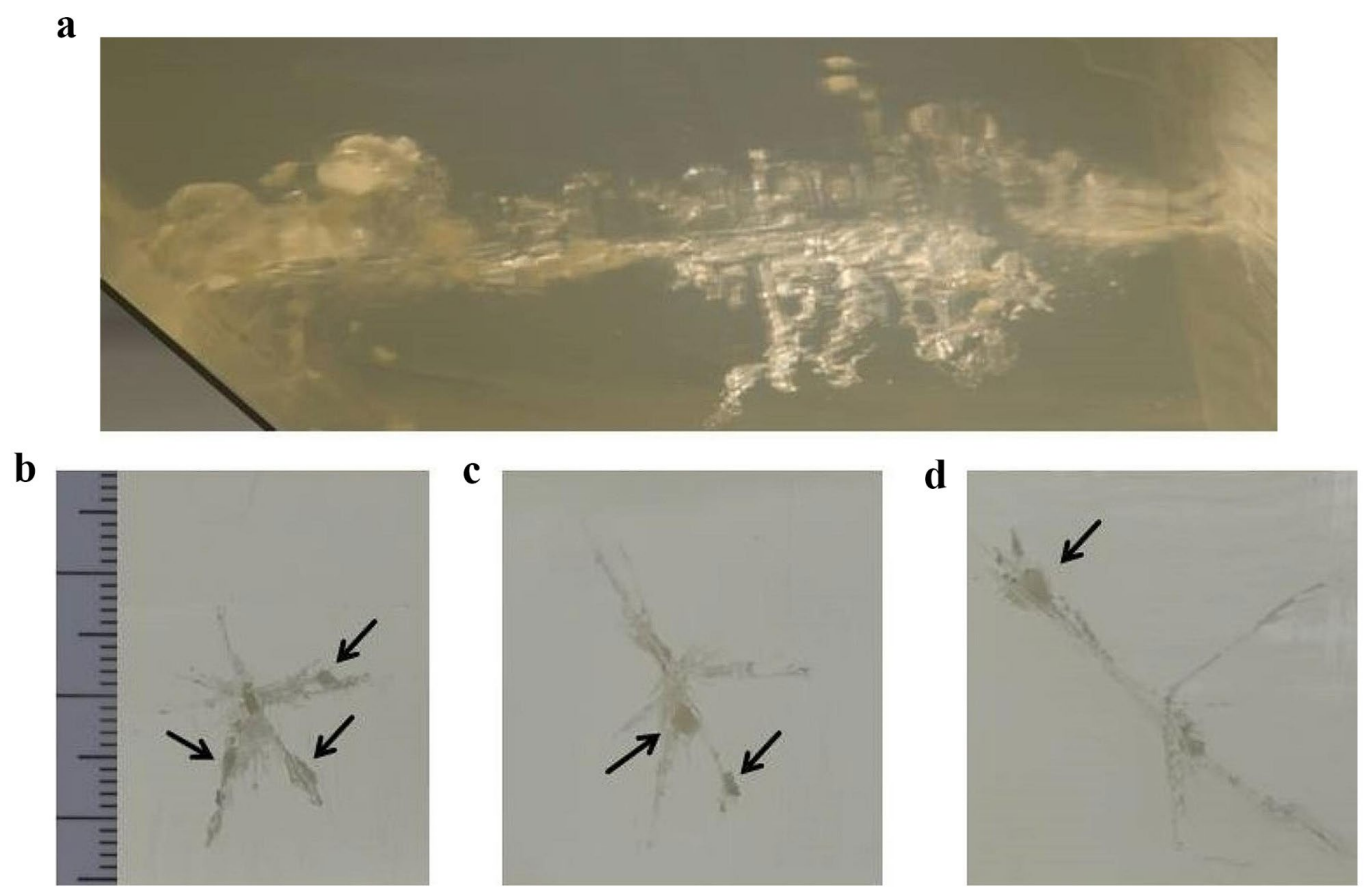
**a** Lateral view to a composite model containing a plate of synthetic bone in the lower left corner. The direction of the shot was from left to right. The cracks in the translucent gelatin indicate the original extension of the temporary cavity. **b** – **d** Transverse sections along the bullet path in gelatin. Small particles of synthetic bone (indicated by *arrows*) are located in the cracks radiating from the permanent wound channel

The final positions of the fragments were also visualized by CT imaging, though artificial bone is less dense compared to osseous tissue of humans (Fig. 2a, b). When cutting the gelatin in layers, the fragments could be localized individually (Fig. 3).

**Fig. 4:**
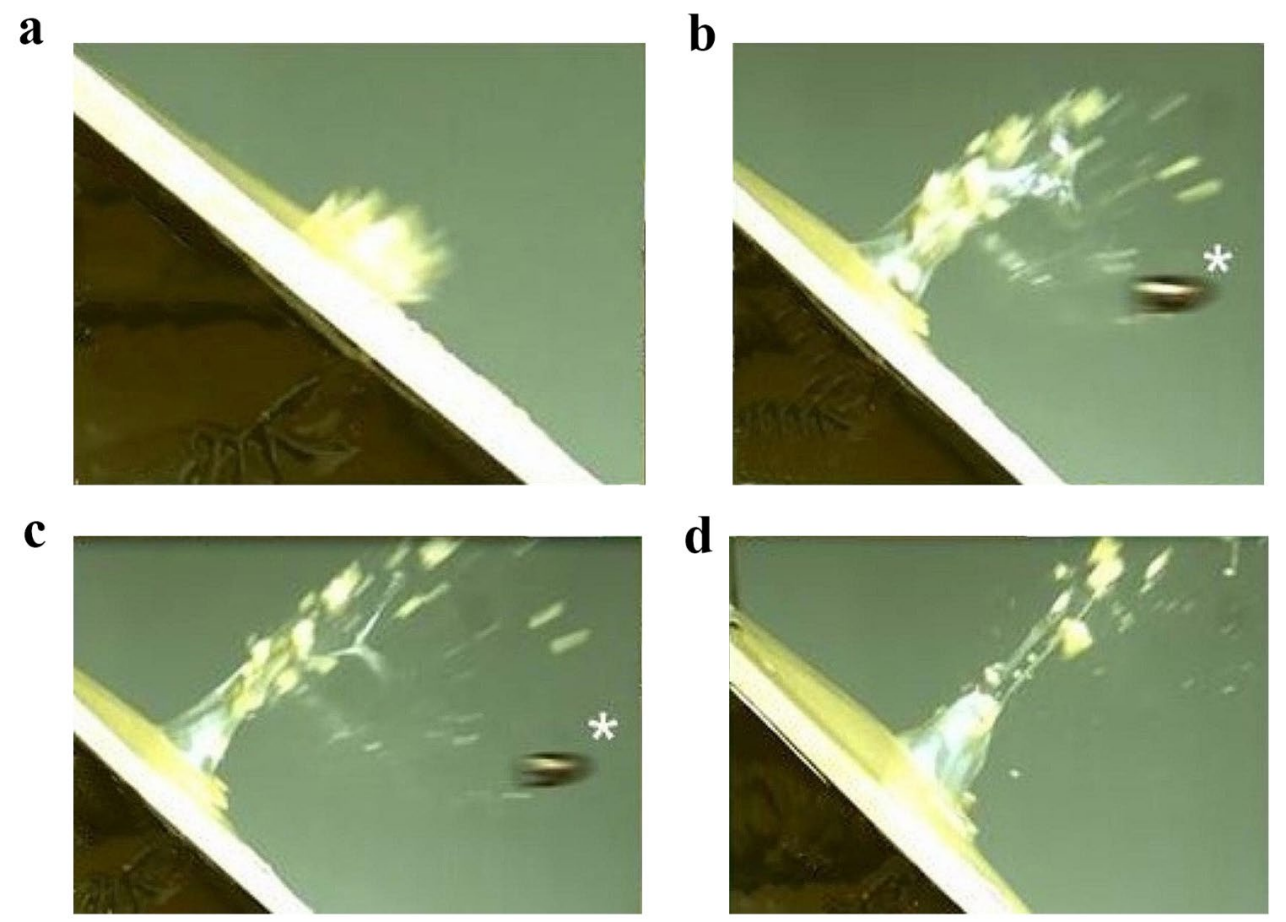
**a** to **d** Short time imaging of a shot to a synthetic bone plate *not* embedded in gelatin: The majority of particles move at right angles to the plate, whereas the projectile (* visible in Fig. 2b and c) is followed by smaller splinters

The additional test shot to a flat bone *not* surrounded by gelatin revealed that the majority of particles, especially the larger ones, travelled at a right angle to the plate, whereas the smaller splinters predominantly moved in the direction of the shot (Fig. 4a–d).

## Discussion

Mutual interaction between bullet and bone may cause a great variety of wound findings often difficult to interpret. Usually, the impacted bone will be perforated and fragmented to a variable degree. In flat bone, the bullet hole is usually roundish on the entrance side and cratered on the opposite side [16, 17]. It was pointed out already in the old literature that the cone-shaped widening on the exit side of bullet holes can give a hint on the direction of the shot [6]. Similar to flat bone, glass panes also show a funnel-shaped widening in the direction of the shot [7]. If the bone is hit at an acute angle, the hole may be elliptic or keyhole-shaped [16] with a corresponding asymmetry of bevelling [18, 19].

For many years, the role of bone particles originating at the perforation site has been a matter of controversial debates. In the past, the bone fragments were thought to act like projectiles [20] and to cause separate wound channels diverging from the actual bullet path [21]. Nowadays, an increasing number of authors do not accept the concept of ‘secondary missiles’: It has been shown experimentally that the bone fragments follow the bullet with some delay and generally move within the dimensions of the temporary cavity. Kneubuehl [11] clearly denies that bone fragments possess enough energy to create separate wound channels deviating from the temporary cavity – “they do not act as secondary projectiles”.

In forensic literature, the special topic of angled shots through bone plates is usually dealt with in the context of keyhole fractures [22, 23]. This type of bone wound is mostly seen in shots to the cranial vault: If the bullet strikes the skull at a shallow angle, the entrance hole in bone will be rounded and sharp-edged on the side facing the shooter and externally bevelled opposite to it. Due to bone fragments breaking out from the outer table and any fragments of the bullet (in cases of disintegration), the wound margin of the skin hole may split unilaterally even in distant shots.

In angled shots through glass panes, the majority of fragments do not follow the projectile’s trajectory, as the mass of splinters move at roughly right angles to the perforated sheet. Accordingly, in a secondary target behind the glass pane the maximum of splinter intrusions will be away from the bullet hole [12, 13]. To the best of our knowledge, up to now no systematic research has been conducted on the distribution of osseous fragments resulting from angled shots to flat bone.

Regarding artificial bone, particular material characteristics should be aimed at [15]: (1) a similar deceleration of the bullet compared with that of human bone; (2) a similar threshold velocity for penetration; (3) a similar fracture behavior. At present, for the training of surgeons as well as for test shots three-layer polyurethane products are used most commonly as they reflect the stratified structure consisting of harder outer and softer inner parts. In our experimental setup, the synthetic bone plate was coated with a thin layer of rubber skin simulating the periosteum.

As described in the methodical section, the test shots were fired to composite models consisting of flat bone surrounded by gelatin. The experimental design did not completely correspond to the skullcap as there was no curvature of the artificial bone comparable with the human anatomy. Composite models have proven useful in many fields of experimental wound ballistics [24–26]. By combining different simulants (gelatin, organs/tissues of slaughtered animals, synthetic bones) complex biological targets can be imitated, albeit in a simplified manner. Major advantages are the potential of standardization and reproducibility.

The ballistic gelatin used in our study was calibrated to an average radiodensity of 35 Hounsfield Units (HU), comparable to human brain tissue. The artificial bone plate had a structural composition comparable to the three-layered skull. However, unlike human bone the radiodensity did not lie in the far positive range between + 300 to + 2000 HU, but rather in the negative range of -200 to -100 for the compact layers and − 350 to -250 for the spongy middle layer (diploë). Therefore, the differences between the radiodensities of artificial bone, gelatin and air are low, thus impeding the discrimination between the components of the composite model, especially in regard to small bone fragments enclosed in gelatin. This discrimination would be much easier with high-radiodensity fragments of real human bone.

The main conclusion drawn from our study is that in angled shots to flat bones most fragments don’t move along the bullet’s path but at appropriate right angles to the bone’s surface plane. This is not surprising as the kinetic energy imparted by the bullet provokes lateral acceleration of the penetrated medium by analogy with the formation of the temporary cavity.

## Conclusions

- In angled shots to flat bones the particles originating at the perforation site behave like splinters from angled shots to glass panes, where the majority of the glass fragments, especially the larger ones, move at right angles to the pane shot through.
- The bone fragments do not act as secondary projectiles.
- Smaller bone fragments are lodged along the bullet’s path, predominantly in the cracks radiating from the permanent wound channel.
- Synthetic bone plates are suitable for shooting test, but it must be taken into account that detection of small fragments in CT scans is difficult because of the simulant’s lower density compared to bone of human or animal origin.

## Data Availability

Data available within the article or its supplementary materials.
